# Thymic pathological examination of non-thymomatous myasthenia gravis patients: A pilot study for prediction of outcome

**Published:** 2014

**Authors:** Zeinab Peimani, Mohammad Amin Banihashemi, Niloofar Namazi, Anahid Safari, Ahmad Monabati, Mehra Mojallal, Afshin Borhani-Haghighi

**Affiliations:** 1 Student Research Comittee, Shiraz University of Medical Sciences, Shiraz, Iran; 2 Research Center for Traditional Medicine and History of Medicine, Shiraz University of Medical Sciences, Shiraz, Iran; 3 Department of Pharmacology, School of Medicine, Islamic Azad University, Kazeroon Branch, Kazeroon, Iran; 4 Department of Pathology, School of Medicine, Shiraz University of Medical Sciences, Shiraz, Iran; 5 Department of Pathology, Dena Hospital, Shiraz, Iran; 6 Health Policy Research Center, Shiraz University of Medical Sciences, Shiraz, Iran; 7 Department of Neurology, School of Medicine, Shiraz University of Medical Sciences, Shiraz, Iran

**Keywords:** Antigens, CD, Bcl-2-Associated X Protein, Immunohistochemistry, Ki-67 Antigen, Myasthenia Gravis

## Abstract

**Background:**

Myasthenia gravis (MG) is an autoimmune disorder characterized by weakness and fatigability of skeletal muscles. The aim of this study was to determine if pathological characteristics in non-thymomatous patients of MG would correlate with prognosis in a three year follow up.

**Methods:**

Patients who had had their thymectomy at least three years prior to the study were selected from three hospitals and were followed for 3 years. Prognosis was assessed via a devised prognostic scoring system. A pathological exam of the specimen from the thymus was done using the following immunohistochemical markers: Bcl2, CD 3, CD 4, CD 5, CD 7, CD 10, CD 20cy, CD 23, CD 43, and Ki67.

**Results:**

Fifteen patients fulfilled the inclusion criteria and had a complete follow-up. This included 3 males and 12 females with a mean age of 36.6 years at the start of the study. The dominant cell population was T lymphocytes. All T cells expressed CD 3, CD 43, CD 5, and Bcl-2. In 2 patients, CD 10 marker was positive in T cells. B cells were negative for activation marker CD 23, except for germinal center dendritic cells. Due to the limited number of patients in the study, the power of the study would not allow for an analysis to assess correlation between histopathological data and prognosis.

**Conclusion:**

This pilot study was an attempt to discover any prognostic indices from the histopathological examination of the resected thymic tissue in the patients with myasthenia gravis.

## Introduction

Myasthenia gravis (MG) is an autoimmune disorder characterized by a defective neuromuscular transmission most probably due to auto-immune pathogenesis.^1^ Thymus is considered as a source of antibody production with about 75% of patients showing thymic abnormalities (85% hyperplasia –germinal center formation and 15% thymomas).^2^

Herein, we studied the pathological thymectomy specimen of non-thymoma MG patients using immunohistochemical (IHC) markers. We tried to find the correlation between IHC findings and prognosis of the patients who were assessed in a 3-year follow-up.

## Materials and Methods

### Study population

The current study was conducted in Faghihi, Namazi, and Dena Hospitals affiliated to Shiraz University of Medical Sciences, Shiraz, south of Iran.

### Inclusion and exclusion criteria

All patients with MG who had had their thymectomy at least three years prior to the study were considered for inclusion in the study via a simple sampling. Diagnosis of MG was made based on clinical and serological testing.^2^

These patients were referred to these centers for thymectomy between 2000 and 2008 and they were followed from 2008 to 2011 prospectively, allowing at least 3 years of follow up for all patients. After explaining the process and the goal of the study for the patients and obtaining informed consents from them, they were referred to a board certified neurologist for a 3-year follow up. Eventually, they were designated either as having a poor or a fair prognosis according to a classification scoring system described below.

### Assessing patient prognosis

Poor prognostic factors were defined as repeated myasthenic crisis, need for recurrent thymectomy, repeated IVIG administration or plasma exchange, need for high dose corticosteroid, and persistent symptoms interfering with daily activities. The follow up and prognostic scoring was done by the same board certified neurologist. This was a double blind study and the neurologist and pathologist were not aware of the other specialist's results.

### Pathological evaluation

Five µm thick sections were cut using a microtome (Leitz 1512 Microtome (WS-LEITZ1512), Ernst Leitz Wetzlar GmBH, Germany) from archival thymectomy specimens of each patient and were stained to evaluate markers [Bcl2 (Dako, Clone 124, Ref. MO887), CD 3 (Novacastra, Ref. NCL-L-CD3-PS1), CD 4 (Novacastra, Clone 4B12, Ref. M7310), CD 5 (Novacastra, Ref RTU-CD5-4C7), CD 7 (Dako, Clone CBC 37, Ref. 7255), CD 10 (Novacastra, Ref. NCL-L-CD10-270), CD 20cy (Dako, Clone L-26, Ref. MO755), CD23 (Novascatra, Ref. RTU-CD23-IB12), CD 43 (Dako, Clone PF-T1, Ref. MO786), Ki67 Antigen (Dako Clone MIB-1, Ref. IR626)] using the Avidin-Biotin-Peroxidase method of immunohistochemistry. Staining was done according to the manufacturer's guidelines. The Envision system was used to label the markers (based on the manufacturer recommendation). For CD 3, CD 4, CD 5, CD 10, and CD 23 the Novacastra Novolink™ Max polymer detection system (Leica Microsystems, Ref. RE7280-K) was utilized. Using this profile the differences in total T and B cell populations, and also the state of B cell activation were determined. Each slide was divided into 100 fields at the high power field level, and subsequently, 10 of those fields were counted randomly.

## Results

Fifteen patients fulfilled the inclusion criteria and had a complete follow-up. This included 3 males and 12 females with a mean age of 36.6 years at the start of the study. Mean duration of MG before thymectomy was 1 year. Eleven patients had a good prognosis and 4 had a poor prognosis. There were no mortalities during the follow up.

Lymphoid elements were the major component of the thymus and only a few of them have been involuted and atrophied by fatty in-growth. The dominant cell population was T lymphocyte, but there were also some B cells present either in lymphoid follicles or diffusely scattered between T cells.

**Figure 1:**
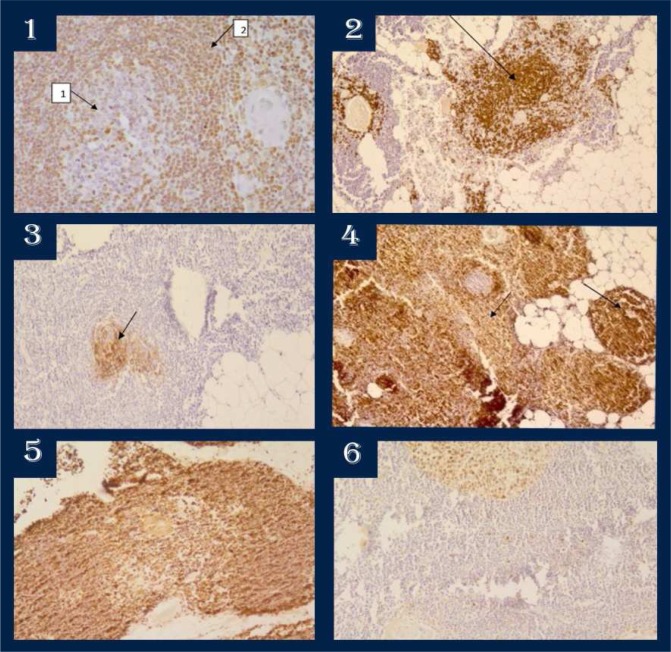
Legend: Section from thymectomy specimens with immunohistochemistry staining 1-1 BCL2-negative cells in normal germinal center (arrow 1), BCL2 positive in T cells (arrow 2) 1-2: CD 20-positive cells in germinal center (arrow) 1-3: CD 23-positive cells seen only in follicular dendritic cells in germinal center (arrow) 1-4: CD 3-positive cells in germinal center and cortical T cells (arrow) 1-5: high Ki67-antigen positive cells 1-6: low Ki67-antigen positive cells

All T cells expressed CD 3, CD 43, and CD 5. All T cells also expressed Bcl-2, which is an anti-apoptotic marker and is usually present in T cells of lymphoid tissues. In 2 patients, CD 10 marker was positive in T cells. This is usually an indicator of a T cell originating from the germinal center (Figure 1).

B cells were negative for activation marker CD 23, except for germinal center dendritic cells. The percentage of cells in proliferation, as measured by Ki-67, varied in lymphocytes outside germinal centers, from as high as 80% to very low. Due to the limited number of patients in the study, the power of the study would not allow for an analysis to assess correlation between histopathological data and prognosis (Table 1).

**Table 1:** Pathological examination and patient characteristics

#	Fat^1^	Lymphoid^2^	CD 20^3^	CD 3^4^	CD 5^4^	CD 43^4^	CD 23^5^	CD 10^6^	Bcl2^7^	Ki67^8^	Prognosis	Age	Gender
1	20	80	30N	70	70	70	N,GC	Positive in T Lymphocytes	Positive	Missing Data	Good	23	F
2	10	90	1	99	99	99	N	Positive in 10% of T Lymphocytes	Positive	40	Good	Missing Data	F
3	20	80	30N	70	70	70	N,GC	Negative	Positive	30	Good	37	F
4	20	80	40	60	60	60	N,GC	Negative	Positive	10	Poor	26	F
5	50	50	40N	60	60	60	N	Negative	Positive	5	Poor	25	F
6	70	30	30N	70	70	70	N,GC	Negative	Positive	80	Poor	48	F
7	30	70	30N	70	70	70	N,GC	Negative	Positive	10	Poor	36	F
8	80	20	50N	50	50	50	N,GC	Negative	Missing Data	Missing Data	Good	50	F
9	40	60	40N	60	60	60	N,GC	Negative	Positive	5	Good	36	M
10	50	50	40N	60	60	60	N,GC	Negative	Positive	5	Good	52	F
11	20	80	30	70	70	70	N,GC	Negative	Positive	80	Good	50	M
12	30	70	30	70	70	70	N	Positive	Positive	70	Good	24	F
13	10	90	20	80	80	80	N	Negative	Positive	50	Good	Missing Data	F
14	30	70	50	50	50	50	N,GC	Negative	Positive	10	Good	46	F
15	30	70	30N	70	70	70	N,GC	Positive in 50% of T Lymphocytes	Positive	80	Good	45	M

1,2- These two columns show the percentage of the thymus tissue occupied by fat deposition or lymphoid tissue. 3-This column shows the percentage of CD 20 positive mature B cells in the thymic tissue. Wherever the B cells have taken the nodular aggregate form this has been shown by including the word “N” in front of the percentage. 4-These columns show the proportion of CD 3-, CD 5-, and CD 43-positive T cells in the thymic tissue. 5-This column shows B cells positive for the CD 23 antigen. Wherever germinal centers were seen, this was marked by “GC”, “N” stands for Nodular Aggregate. 6-The CD 10 marker normally present in germinal centers is shown here. The percentage of CD 10 expression in cases 1, 2, 12, and 15 were ∼100%, 10%, ∼100%, and 50%, respectively. 7-The Percentage of cells showing BCL2 expression outside germinal centers. 8-The Percentage of cells showing Ki67 expression as a marker of proliferation

## Discussion

Myasthenia Gravis may have an unpredictable course after thymectomy; therefore, histopathological criteria can be a useful tool for predicting the course of the disease to aid in choosing an appropriate therapeutic regimen. Despite recent advances, assessing prognosis is not an easy task as there are not many reliable indicators of outcome. A systematic review, which included 19 variables from 13 studies, showed inconclusive evidence for the majority of assessed prognostic factors; none showed an assessment of IHC factors.^3^ The only factor which was predictive of remission was diagnosis under one year from the onset of symptoms. In one of the studies in this review, a histological diagnosis of thymic hyperplasia was significantly associated with complete stable remission.^4^ In a more recent study, pathological non-immunohistochemical findings were not statistically significant.^5^

We specifically assessed surface markers by means of IHC, something that is not frequently reported in association with prognosis. Some of these markers have been shown to play a role in the pathogenesis of MG. We could not analyze the data to look for a correlation between histopathological markers and prognosis as there were too few patients.

This study's most important shortcoming was the low number of available patients and the substantial number of missed follow-up visits of patients, which contributed to a lower than expected sample size, resulting in an inability to analyze the data obtained.

## Conclusion

Prognostication and tailoring of appropriate therapeutic options for patients with MG who undergo thymectomy is of great importance.

Considering the lack of reliable prognostic indicators for MG patients, we conducted the current study to assess whether immunohistopathological features of the thymic specimens could predict prognosis. Although we did not reach statistical significance due to a small sample size, we shared the idea and showed technical feasibility in the current study. Therefore, similar studies in large-volume referral centers are recommended.
